# People-centred surveillance: a narrative review of community-based surveillance among crisis-affected populations

**DOI:** 10.1016/S2542-5196(20)30221-7

**Published:** 2020-10-07

**Authors:** Ruwan Ratnayake, Meghan Tammaro, Amanda Tiffany, Anine Kongelf, Jonathan A Polonsky, Amanda McClelland

**Affiliations:** a International Rescue Committee, New York, NY, USA; b Department of Infectious Disease Epidemiology, London School of Hygiene & Tropical Medicine, London, UK; c Johns Hopkins Bloomberg School of Public Health, Johns Hopkins University, Baltimore, MD, USA; d Epicentre, Geneva, Switzerland; e Norwegian Red Cross, Oslo, Norway; f World Health Organization, Geneva, Switzerland; g Institute of Global Health, Faculty of Medicine, University of Geneva, Geneva, Switzerland; h International Federation of Red Cross and Red Crescent Societies, Geneva, Switzerland

## Abstract

Outbreaks of disease in settings affected by crises grow rapidly due to late detection and weakened public health systems. Where surveillance is underfunctioning, community-based surveillance can contribute to rapid outbreak detection and response, a core capacity of the International Health Regulations. We reviewed articles describing the potential for community-based surveillance to detect diseases of epidemic potential, outbreaks, and mortality among populations affected by crises. Surveillance objectives have included the early warning of outbreaks, active case finding during outbreaks, case finding for eradication programmes, and mortality surveillance. Community-based surveillance can provide sensitive and timely detection, identify valid signals for diseases with salient symptoms, and provide continuity in remote areas during cycles of insecurity. Effectiveness appears to be mediated by operational requirements for continuous supervision of large community networks, verification of a large number of signals, and integration of community-based surveillance within the routine investigation and response infrastructure. Similar to all community health systems, community-based surveillance requires simple design, reliable supervision, and early and routine monitoring and evaluation to ensure data validity. Research priorities include the evaluation of syndromic case definitions, electronic data collection for community members, sentinel site designs, and statistical techniques to counterbalance false positive signals.

## Introduction

Humanitarian crises and forced migration disrupt public health systems and increase the risk of the emergence and transmission of infectious diseases.^1^, ^2^ Outbreaks can reach widespread geographical scales when undetected by surveillance and transmission is unmitigated by public health systems. The direct effect can be substantial morbidity and mortality; examples include the Ebola virus disease outbreaks in west Africa in 2014–16 and the Democratic Republic of the Congo from 2018 to 2020, and an ongoing cholera outbreak in Yemen, which began in 2016.^3^, ^4^, ^5^ A review of delays in detection in fragile states (ie, a country that has severely weak state policies and institutions or is affected by ongoing or past violent conflict, or both), reported a median delay of 29 days (range 7–80 days) from the onset of symptoms of the index case to outbreak detection.^6^ This period could encompass multiple generation intervals of many diseases of epidemic potential, including cholera, Ebola virus disease, and shigellosis, presenting a clear risk of large-scale disease expansion before the initial clusters are detected.^7^

To improve the early detection of outbreaks and to sustain disease surveillance in crises, early warning, alert, and response systems (EWARS) are recommended.^8^ In crises, where routine surveillance is partly functional, non-functional, or does not cover the whole population, community-based surveillance (CBS) is often applied to detect events of concern to public health.^9^ CBS has been defined by a WHO interagency working group as “the systematic detection and reporting of events of public health significance within a community by community members”.^10^ A combination of existing networks of community health workers (CHWs), Red Cross society volunteers, and newly recruited key informants typically constitutes the workforce of any CBS system.^11^, ^12^ The community under surveillance is defined according to the context,^10^ but generally covers the catchment area that is physically accessible to the workforce and considered to be socially cohesive.

**Key messages**

• Outbreaks in settings affected by crises grow rapidly due to late detection and weakened surveillance and public health systems.
• Community-based surveillance in humanitarian crises was investigated to understand its potential for detecting and monitoring diseases of epidemic potential, outbreaks, and mortality among populations affected by crises.
• Nearly all community-based surveillance systems showed that community members can signal diseases with salient symptoms. Community-based surveillance can also provide timely and sensitive detection of cases and outbreaks, and continuous surveillance during periods of insecurity.
• Key challenges involved assuring efficiency through continuous supervision for large community networks, availability of resources to investigate and verify a high volume of signals, and integrated verification and response with the existing infrastructure.
• Crucial areas for future study include documentation of early and routine evaluation to improve data validity, use of sentinel site designs, and adoption of electronic data collection methods to enable rapid implementation and analysis.

Through integrated disease surveillance and response strategies of ministries of health, forms of CBS have started in 32 of 47 countries in the WHO African region.^13^ In crises, CBS can be facilitated by non-governmental organisations to aid in providing early warning of deteriorations in public health, active case finding during outbreaks, and for surveillance coverage in displacement camps. Eradication programmes in fragile settings use CBS to increase the sensitivity of detection of poliomyelitis and Guinea worm in remote areas (eg, the CORE Group Polio Project).^14^

Despite increasing recognition of the importance of CBS in settings affected by crises, there has been little research into standard approaches, optimal implementation strategies, and its effectiveness.^9^ We reviewed studies on the potential for CBS to detect epidemic-prone diseases, outbreaks, and mortality among populations affected by crises. We also summarised the effectiveness of CBS for outbreak detection and response.

## Literature review

**Figure 1:**
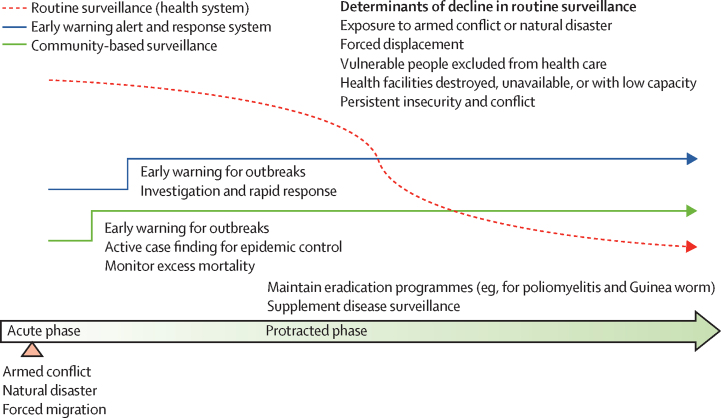
Conceptual framework for surveillance systems in crises

The literature search was guided by a conceptual model for CBS among populations affected by crises that outlines gaps in surveillance in health facilities and the capacities of EWARS and CBS throughout the acute and protracted phases of crises (figure 1). References of retrieved articles and an additional scoping review^9^ were searched. Eligible countries were defined by use of the World Bank's list of fragile and conflicted-affected situations.^15^ Such countries are characterised by violent conflict of a medium to high intensity or a high level of fragility within their institutions or society, or both.^15^ Countries appearing on the list for at least 6 of the 12 years between Jan 1, 2008, and Dec 31, 2019, were eligible (appendix p 1). This search focused on the countries that were fragile or affected by conflict, or both. To expand the inclusion criteria to reflect the diversity of humanitarian settings, a further non-country specific search enabled inclusion of CBS systems in countries that host refugee camps (eg, Jordan, Kenya, and Uganda) or have subnational crises, but are not considered to be fragile by the World Bank (eg, extreme north of Cameroon).

Outcomes broadly included variables describing implementation and, if an evaluation was done, surveillance effectiveness (eg, timeliness, sensitivity, positive predictive value, feasibility, and acceptability).^16^ Two reviewers (RR and MT) screened abstracts and papers for full-text review. A quality assessment of included studies was not done, but studies were excluded on the basis of apparent low quality of analysis,^17^ and major concerns about the methods in included studies were noted.

Data were extracted by two reviewers (RR and MT) and evaluated against a standardised form to capture implementation variables, strategies, and effectiveness outcomes (panel). Synthesis entailed the review of implementation and outcomes, including crisis phase (eg, acute [≤2 months after displacement or natural disaster], protracted, or other fragile setting) and setting (eg, camp, rural or remote, urban, or mixed rural or urban), design (eg, exhaustive or sentinel site surveillance; data collection strategies), objectives (eg, supplementing disease surveillance or EWARS, active case finding, mortality surveillance, or eradication programmes), and evaluation outcomes (when available).

Panel **Descriptive measures and outcomes extracted from studies**

• Country, area, and dates of operation
• Crisis (ie, acute phase, protracted phase, or other fragile setting) and setting (ie, camp, rural or remote, urban, or mixed rural or urban)
• Objectives of the surveillance system (ie, supplement disease surveillance or early warning, alert, and response systems; outbreak response; mortality surveillance; or eradication programme)
• Intended scale and geographical coverage
• Diseases or events, or both, under surveillance
• Format of reports (eg, suspect case, death report, birth report, etc)
• Reporter (ie, community health worker or volunteer); renumeration or incentives; training or supervision
• Frequency of disease reporting
• Means of transmitting data; technology or software
• Data flow
• Facilitating or stakeholder organisations involved
• Stability and sustainability
• Public health response to alerts
• If evaluation was done:
  • Attributes evaluated and results (eg, timeliness, sensitivity, positive predictive value, simplicity, and acceptability)
  • Quality of analysis

## Use of CBS systems

**Figure 2:**
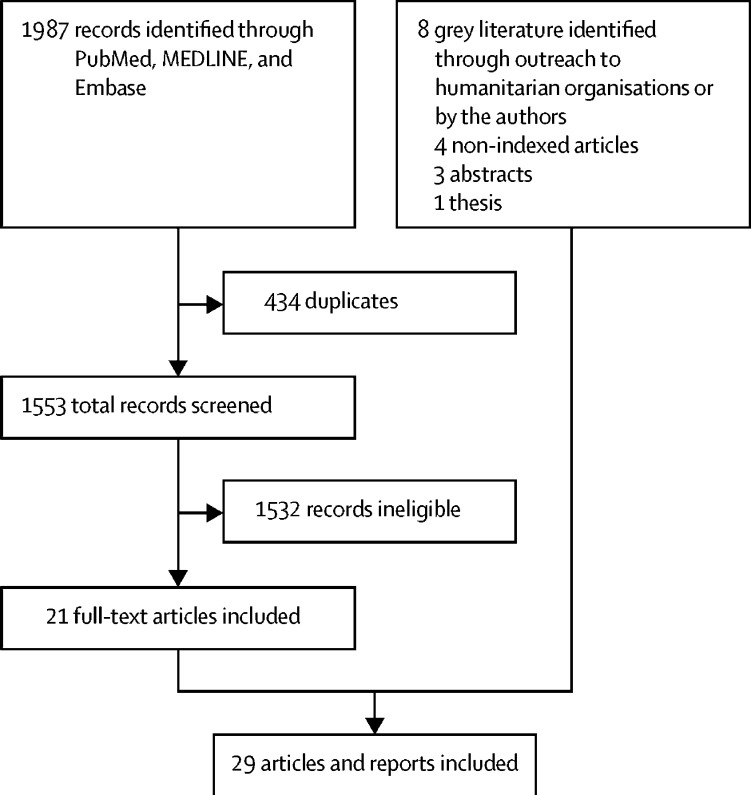
Flow diagram of the search strategy

The search produced 1987 unique results and 21 articles that met the inclusion criteria (figure 2).^18^, ^19^, ^20^, ^21^, ^22^, ^23^, ^24^, ^25^, ^26^, ^27^, ^28^, ^29^, ^30^, ^31^, ^32^, ^33^, ^34^, ^35^, ^36^, ^37^, ^38^ Eight documents were provided by humanitarian organisations (three abstracts,^39^, ^40^, ^41^ two non-indexed articles,^42^, ^43^ and one thesis^44^) or by the authors (one older, non-indexed article^45^ and one article that did not have MeSH keywords^46^). The 29 documents retrieved^18^, ^19^, ^20^, ^21^, ^22^, ^23^, ^24^, ^25^, ^26^, ^27^, ^28^, ^29^, ^30^, ^31^, ^32^, ^33^, ^34^, ^35^, ^36^, ^37^, ^38^, ^39^, ^40^, ^41^, ^42^, ^43^, ^44^, ^45^, ^46^ described 25 unique systems in the Central African Republic (two systems),^21^, ^33^ Chad,^35^ Côte d’Ivoire (two systems),^24^, ^40^, ^43^ the Democratic Republic of the Congo (two systems),^26^, ^30^ Guinea,^37^ Haiti (two systems),^31^, ^39^ Liberia,^44^ Sierra Leone,^25^, ^32^, ^36^, ^42^ South Sudan (two systems),^27^, ^29^ and Yemen,^46^ and in refugee camps in Bangladesh,^41^ Chad,^19^ the Democratic Republic of the Congo,^22^ Ethiopia,^20^ India,^18^ Malawi,^45^ Nepal,^38^ South Sudan,^23^ Tanzania,^28^ and Uganda^34^ (table 1). Study designs included descriptive studies of outbreak responses (16 of 29 studies; seven studies had only basic details on CBS)^20,22,23,25,27,28,30,31,33–35,38,40,42,43,46^ and evaluations of CBS performance (13 of 29 studies).^18^, ^19^, ^21^, ^24^, ^26^, ^29^, ^32^, ^36^, ^37^, ^39^, ^41^, ^44^ The results are reported by use of the denominators for the total number of unique systems (n=25), or the surveillance objective.

**Table 1:** Community-based surveillance systems in alphabetical order of country

	**Disease or events**	**Purpose**	**Design, staffing, investigation, and response**	**Evaluation results**
**Bangladesh**				
13 subcamps in Cox's Bazaar refugee complex (n=548 739);^41^ May–November, 2019	Acute flaccid paralysis, acute watery diarrhoea, acute jaundice syndrome, diphtheria, measles, meningitis, dengue, mortality	Early warning for outbreaks; monitor community mortality trends (notably neonatal deaths; camp setting)	CHVs did active surveillance, covering on average 36 households per day; no information on training and supervision; CBS had integrated alert and response team and medical response team to launch rapid response to CBS alerts; the system was integrated into early warning alerts and response system	Comprehensive evaluation; multiple rapid diagnostic tests and cholera alerts triggered a targeted cholera response in surrounding households; high PPV for acute flaccid paralysis, acute watery diarrhoea, and measles (74–100%) and low PPV for meningitis and diphtheria (42–50%); resource intensive, requiring 354 staff, including alert and medical response teams
**Central African Republic**				
24 villages, Boda, Boganangone, Boganda, and Gadzi subprefectures (n=158 000);^21^ March–December, 2010	Child and crude mortality	Monitor health trends in a crisis (rural setting)	24 CHVs did active surveillance once per week for crude mortality and migration in or out of the setting; 24 sentinel sites were randomly selected	Evaluation of sensitivity and data validity; there was low attrition and CBS was acceptable to communities; capture–recapture analysis showed that mortality data had >90% sensitivity and specificity; population data were difficult to estimate because of high migration in or out of the setting; sentinel site CBS is feasible in a rural crisis setting
80 villages, Paoua (Ouham-Pendé) and Markounda (Ouham) subprefectures (n=222 000);^33^ 2009–14	Malaria (suspect cases)	Monitor malaria trends in a crisis (rural setting; acute phase)	80 CHWs did case management of malaria among children <5 years and pregnant women in the community and reported surveillance data once per month	No formal evaluation; CHWs maintained malaria surveillance during and after a crisis where no other sources of surveillance existed (near 100% reporting rates); CHWs migrated with villages; the assumption of constant population size is most likely untrue; high costs of supervision and training can only be supported by a non-governmental organisation
**Chad**				
Five refugee or internal displacement camps in eastern Chad (ie, Farchana, Breidjing, Ade, Gassire, and Kerfi; n=13 000–30 000);^19^ 2004–08	Child and crude mortality	Monitor mortality trends in a crisis (camp setting; acute phase)	CHWs (n=unknown) did active surveillance for births, deaths, and migration, collated once per week; CHWs assigned to 500 households each	Evaluation of validity, simplicity, flexibility, and timeliness; CBS helped to detect a diarrhoea outbreak that led to improvements in water quality; lessons learned included the need for improved population estimates, standardised reporting, and procedures for improved data quality and dissemination, the importance of a simple and flexible model for data collection, and supervising CHWs; CBS implementation too slow for emergency phase (2–5 months to implement)
Am Timam town, eastern Salamat region (n=65 000);^35^ September, 2016, to April, 2017	Hepatitis E (acute jaundice syndrome)	Active hepatitis E surveillance during an epidemic (periurban setting)	160 CHVs visited households twice a week to do surveillance of acute jaundice syndrome and immediate referral for people at risk of clinical complications	No formal evaluation; CBS made difficult by high mobility of population (seminomadic herders); might have underestimated true number of cases
**Côte d'Ivoire**				
Biankouma, Danané, and Zouan-Hounien districts, Tonkpi region (n=992 565);^40^ March–May, 2015	Ebola virus disease	Active Ebola virus disease surveillance during an epidemic (rural setting)	110 CHWs reported events suggestive of Ebola virus disease every day, including deaths, animal contacts, and illness or deaths among health workers and visitors; FrontlineSMS was used to transmit data immediately	No formal evaluation
Odienné, Touba, and Minignan districts, Kabadougou-Bafing-Folon region (n=501 328);^24^, ^43^ April, 2016, to December, 2017	Polio, cholera, measles, meningitis, yellow fever, and unusual health events	Early warning of outbreaks in a border area where Ebola virus disease was present	541 CHWs and a key informant network visited homes and were notified of signals; they used FrontlineSMS to immediately transmit signals; triage nurse received signal and carried out initial investigation	Evaluation of effectiveness; large-scale increases in reporting of suspect cases after implementation; low yield of nurse-verified suspect cases (11% [420 of 3734]) and confirmed cases (5% [23 of 420]); highest proportion of suspect cases for polio (49% [33 of 68]) and yellow fever (29% [166 of 568]); cholera produced no verified suspect cases but 1857 signals, producing a large burden on the system; large burden of human resources, supervision, and costs
**Democratic Republic of the Congo**				
Rwandan refugee camps, Goma (n=90 000);^22^ April–May, 1992	Cholera	Active cholera surveillance during an epidemic in a crisis (camp setting)	CHWs (n=unknown) did active case finding of cholera cases (defined as people with sudden onset of watery diarrhoea resulting in dehydration)	No formal evaluation
39 health areas, Katanga Province;^30^ population unknown; April–May, 2011	Measles and measles-related mortality	Active measles surveillance during an epidemic (rural setting)	CHVs (n=unknown) interviewed community leaders about suspected measles-related deaths (within 30 days of onset of rash and not due to an obvious other cause, such as trauma) and collected information on measles deaths once per week	No formal evaluation; CBS reported higher numbers of measles-related deaths (n=376) *vs* facility surveillance (n=27); authors reported minimal time and resources were put into CBS in this context
34 health centre catchment areas, Fizi Health Zone, South Kivu Province (n=2926);^26^ October, 2011, to September, 2012	Crude mortality	Monitor mortality trends in a crisis (rural setting)	CHVs (n=unknown) did active surveillance once per month at preselected households on deaths (crude) and births in the past month while accounting for population changes; 34 sentinel sites were selected non-randomly from Fizi Health Zone and cluster sampling was used to select 15 households in each cluster	Evaluation assessed data validity, sensitivity, specificity, PPV, simplicity, and flexibility; by use of a mortality survey and resolution of differences in recorded births and deaths between surveillance and the survey, the evaluation reported improved sensitivity (87% *vs* 71%), specificity (>99% *vs* 99%), and PPV (>91% *vs* 28%) for surveillance over the survey; data collection was simple and flexible, as it was reduced to few data elements and visits could be delayed due to insecurity; surveillance needed maintenance and multiple layers of data checking
**Ethiopia**				
Three South Sudanese refugee camps, Gambella (n=approximately 150 000);^20^ April, 2014, to January, 2015	Hepatitis E	Active hepatitis E surveillance during an epidemic in a crisis (camp setting)	CHWs (n=unknown) detected and referred acute jaundice syndrome cases to health facilities	No formal evaluation
**Guinea**				
Three subprefecture of Guéckédou prefecture (Guéckédou city, Tékoulo, and Guendembou; n=43 000);^37^ 2011–14	Child and crude mortality and causes (malaria and Ebola)	Monitor trends in malaria mortality (rural setting)	46 CHVs did passive surveillance of deaths and suspected causes (using a simple algorithm to attribute to malaria, Ebola, or other cause) in 46 sentinel sites; one CHW to every 12 500 people	Evaluation assessed validity of mortality data; CBS can capture information on mortality in areas where surveillance is weak or patients do not present to facilities (eg, during Ebola virus disease outbreak); establishing causes of death is challenging; CBS of mortality is useful for outbreak detection if timeliness of data collection and reporting facilitate real-time data analysis
**Haiti**				
Internal displacement camps in Delmas (n=43 930–54 890) and Champs de Mars (n=approximately 23 500), Port au Prince;^31^ epidemic weeks 12–32, 2010	Child and crude mortality	Monitor health trends during a crisis (urban setting; acute phase)	CHVs (n=unknown) assessed child and crude mortality, births, and migration in or out of the setting in households in a week	No formal evaluation; CBS recorded lower than anticipated mortality rates (with the assumption that mortality was very high following the earthquake) and shifted focus toward other needs; communities were frustrated that visits once per week did not yield immediate benefits; threats toward home visitors caused closure of CBS; high migration in or out of the setting; denominators were difficult to establish
Western area;^39^ population unknown; 2014–15	Cholera	Active cholera surveillance during an epidemic (rural setting)	239 Red Cross CHVs did active surveillance for cholera (acute watery diarrhoea) every day by use of Magpi or SMS on a mobile phone; suspect cases were referred immediately and logged	Evaluation of sensitivity and specificity; CBS appeared sensitive (ie, it compared well over time with facility data) and detected cholera in geographical areas not covered well by facility surveillance; CBS had high sensitivity with high numbers of false positives; CBS should be used with facility surveillance (low sensitivity, high specific) to show the overall perspective
**India**				
37 Tibetan refugee settlements and Delhi (South, Doon Valley, Central, Ladakh, Himachal, and North East; n=54 537);^18^ 1994–96	Child and crude mortality, causes of death	Monitor health trends during a crisis (urban setting; rural setting, long-term)	CHWs (n=unknown) collected monthly data for mortality and cause of death from households of refugees in India; CHWs sent data by paper to a central office in Dharamsala	Evaluation of various aspects (largely qualitative); CBS provided a profile of cause of death but unrealistically low death rates; CHW visits once per month were not always possible as CHWs were overworked; therefore, collection of monthly morbidity data was not possible, as was intended
**Liberia**				
43 districts in Grand Cape Mount, Gbarpolu, Lofa, Bong, Nimba, Grand Gedeh, River Gee, and Maryland (n=2 million);^44^ February–October, 2016	Acute flaccid paralysis, measles, rabies, acute bloody diarrhoea, meningitis, viral haemorrhagic fever, acute watery diarrhoea, neonatal tetanus, death (neonatal and maternal), and unexplained cluster (disease or death)	Early warning of outbreaks in a border area where Ebola virus disease is present	2972 surveillance volunteers (1:100 population) were notified of potential cases or events that would be referred to the health facility; integrated disease surveillance and response guidelines were followed to investigate and notify the suspect case	Evaluation of effectiveness; 24% (885 of 3746) of alerts were suspect cases according to the community case definition; 32% of non-Ebola virus disease cases of epidemic disease were signalled by CBS; PPV was highest for neonatal deaths (70% [50 of 71]), maternal deaths (82% [27 of 33]), and unexplained deaths (43% [50 of 117]) and low for viral haemorrhagic fever (8% [40 of 505]), meningitis (10% [13 of 125]), and acute flaccid paralysis (14% [10 of 71]); coverage among CBS and health facilities was highest for acute watery diarrhoea, neonatal tetanus, acute flaccid paralysis, and unexplained death; CBS was financially unsustainable (19% of government health expenditure)
**Malawi**				
11 Mozambican refugee camps (n=269 859);^45^ 1987–89	Child and crude mortality, causes of death	Monitor mortality and cause of death trends in a crisis (camp setting; acute phase); relied on health posts for morbidity surveillance	CHWs in 11 sections counted deaths each week in sections of up to 2000 persons; causes of death were investigated by use of health-facility registries and interviews with family members, where case definitions were used to assign a cause of death; the goal was to identify diseases related to the highest cause of mortality in the refugee camp	Qualitative evaluation of simplicity, flexibility, and adaptability; a basic evaluation was done of both CBS and health facility-based surveillance (morbidity); the entire surveillance system was assessed to be simple and acceptable on the basis of the few steps and sources (ie, CHWs, health posts) used, use of the data collection infrastructure of the ministry of health, and rapid weekly reporting
**Nepal**				
Six Bhutanese refugee camps, Teraj region (n=73 500);^38^ July, 1992, to January, 1993	Child and crude mortality, causes of death	Monitor mortality and cause of death trends during a crisis (camp setting; acute phase); relied on health posts for morbidity surveillance	One CHV specialising in mortality per camp did active surveillance every day of deaths and causes of deaths (by use of a simple algorithm for malaria, measles, acute respiratory illness, diarrhoea, death in childbirth, injury, or other or unknown); the data collection approach was not described; on the basis of mortality data, CHWs later focused on active case finding of acute respiratory illness and diarrhoea to provide immediate treatment and referral	No formal evaluation; CBS led to public health actions and rapid detection and response to cholera, *Shigella dysenteriae,* and meningoencephalitis; provision of burial expenses encouraged reporting from the community
**Sierra Leone**				
Bo, Kailahun, Kambia, Kenema, Kono, Moyamba, Pujehun, and Tonkolili Districts (nine of 14 districts; n=3 981 665);^25^, ^32^, ^36^ Feb 27 to Sept 30, 2015	Ebola virus disease (events)	Active Ebola virus disease surveillance during an epidemic (mostly rural setting)	CBS based on events was rapidly scaled at a national level by a network of non-governmental organisations during a health emergency; 7416 CHWs and CHVs and 137 supervisors did active surveillance every day of six events suggestive of Ebola virus transmission, including community deaths, by use of simple phones	Evaluation of sensitivity and timeliness; of the 12 126 reports, 287 reports (2%) met the suspected case definition, 16 reports were confirmed positive (detecting 30% [16 of 53] of Ebola virus disease cases identified during the study period); consistent surveillance data was produced from districts reporting few or no cases; CBS detection was faster than facility detection and new chains of transmission were found; CBS cost US$1·3 million at start-up with approximately $129 000 monthly costs; to sustain performance, event definitions should be refined and integrated into the surveillance system
**South Sudan**				
National scale, population not provided;^27^ 22 000 villages; 2006–12	Guinea worm	Eradication (case containment) in a crisis (mostly rural setting)	CHWs (n=unknown) did active surveillance every day at village level and implemented case containment	No formal evaluation
Yusuf Batil, Jamam, Gendrassa, and Doro refugee camps, Maban County, Upper Nile (n=110 000);^23^ July, 2012, to January, 2013 (7 months)	Hepatitis E (acute jaundice syndrome)	Active hepatitis E surveillance during an epidemic in a crisis (camp setting)	CHWs (n=unknown) detected and referred cases of acute jaundice syndrome to health facilities	No formal evaluation
34 counties, Jonglei, eastern Equatoria, Unity State, and Upper Nile states (n=3·7 million);^29^ October, 2015, to September, 2017	Acute flaccid paralysis	Eradication (case containment) in a crisis (mostly rural setting)	3228 community key informants passively reported cases to 230 payam assistants; key informants given training in case identification; payam assistants visited key informants once per week; suspect case triggered WHO field team to do specimen collection	Evaluated functionality, sensitivity, and effectiveness; counties that regularly reported increased from 64% (16 of 25) to 92% (23 of 25); increase in acute flaccid paralysis reporting from CBS (12·5 cases per month) *vs* health facilities (2·2 cases per month); CBS incurred a low stool sampling rate (51–63%) compared with 100% in health facilities due to difficulties tracing suspect cases and a scarcity of specimen sampling materials and transport; higher proportions of cases reported within 24 h and investigated within 48 h with CBS than with health facilities, but the denominators used were unclear
**Tanzania**				
Four refugee camps in Kibondo District (n=279 455);^28^ March, 2000, to May, 2001 (1 year, 3 months)	Measles	Active measles surveillance during an epidemic in a crisis (camp setting)	CHWs (n=unknown) did active case finding in households on five diseases of epidemic potential, including measles; two surveillance focal people reconciled CHW-identified suspect cases and health facility registers to identify missed measles cases	No formal evaluation; authors state that “this epidemic was remarkable for its scarcity of reported measles-associated deaths despite rigorous community-based surveillance”^28^
**Uganda**				
East Moyo South Sudanese camp (n=30 000);^34^ February, 1994, to March, 1995 (1 year)	Meningococcal meningitis	Active meningitis surveillance during an epidemic in a crisis (camp setting)	CHWs (n=unknown) did active surveillance and referral for meningitis cases	No formal evaluation
**Yemen**				
Al Hawak and Al Mena districts, Hodeidah City (n=400 000);^46^ October, 2016, to February, 2017 (4 months)	Cholera	Active cholera surveillance during an epidemic in a crisis (urban setting)	Community teams monitored households in neighbourhoods where cholera suspect cases came from to do active case finding for cholera (acute watery diarrhoea); mild cases were given oral rehydration solution and complicated cases were referred	No formal evaluation

CBS=community-based surveillance. CHV=community health volunteer. CHW=community health worker. PPV=positive predictive value.

CBS was implemented during acute phases (six of 25 systems; eg, postearthquake Haiti and recent displacement in the Democratic Republic of the Congo and Bangladesh), protracted phases (13 of 25 systems; a mix of long-term camps and protracted crises—eg, conflict in the Democratic Republic of the Congo), and other fragile settings (six of 25 systems; eg, Ebola virus disease and post-Ebola virus disease in Sierra Leone, Liberia, and Côte d’Ivoire). Intervention sites included rural and remote settings (ten of 25 systems), refugee or internal displacement camps (ten of 25 systems), and urban or mixed settings (five of 25 systems). The largest systems were scaled to nearly national levels to detect Ebola virus disease events in Sierra Leone (with a target population of 3·9 million people), acute flaccid paralysis in South Sudan (with a target population of 3·7 million people), and multiple epidemic-prone diseases in Liberia after outbreak of Ebola virus disease (with a target population of 2·1 million people).^29^, ^32^, ^44^ The median target population sizes were 222 000 people (IQR 81 292–225 714) in acute phases, 190 000 people (IQR 110 000–311 295) in protracted phases, and 269 859 people (IQR 328 811–1 674 600) in fragile settings.

The main objectives of CBS included providing support to health facility-based surveillance or EWARS for active case finding during large outbreaks (ten of 25 systems) or mortality surveillance to detect deteriorations in health status typically due to endemic communicable diseases and acute malnutrition (ten of 25 systems); early detection of outbreaks (six of 25 systems); and eradication programmes (two of 25 systems; Guinea worm and poliomyelitis, both in South Sudan). In acute phases, all systems monitored mortality trends.^19^, ^22^, ^31^, ^38^, ^41^, ^45^ In protracted phases or fragile settings, CBS systems were predominantly used for outbreaks of a single disease,^20^, ^28^, ^35^, ^36^, ^39^ or for early warning of outbreaks of multiple epidemic-prone diseases.^24^, ^28^, ^44^ Eradication programmes maintained passive detection of suspected cases of poliomyelitis and Guinea worm during interspersed periods of insecurity and relative stability in South Sudan.^27^, ^29^ Guinea worm detection also included isolation and treatment by community health volunteers (CHVs).^27^

Nearly all systems (22 of 25) used an exhaustive design over a continuous geographical area. Sentinel site designs that used randomly selected and representative sites were used to monitor mortality trends (three of 25 systems).^21^, ^26^, ^37^ Sentinel designs required fewer personnel and resources but needed support from epidemiologists throughout the design and implementation period.^21^, ^26^, ^37^ The CBS workforce included existing CHWs and CHVs from networks supported by ministries of health or non-governmental organisations (15 of 25 systems), Red Cross society volunteers (three of 25 systems), or newly recruited surveillance volunteers or key informants (nine of 25 systems). To reach scale for large systems in Sierra Leone and Côte d’Ivoire, a mix of existing CHWs and newly recruited surveillance volunteers was used.^24^, ^32^ Few details on training and supervision plans (eight of 25 systems) and financial and material incentives (eg, rain gear; four of 25 systems) were given.

The ratio of the workforce to the population under surveillance varied considerably depending on the system design and method of data collection (table 2). Sentinel systems for mortality in the Central African Republic, Guinea, and the Democratic Republic of the Congo had low proportions of workforce to households (approximately 1:1000–1500) given the requirement to visit only sampled sites and households within the catchment area.^21^, ^26^, ^37^ By contrast, to exhaustively monitor mortality in camps in Chad with existing CHWs, a ratio of 1:83 was applied.^19^ Low-intensity monitoring was used in six camps in Nepal, where one reporter per camp (1:2042), who also distributed funeral shrouds at no cost, was passively notified of deaths and then investigated the cause of death.^38^ Among CHWs treating and surveilling children younger than 5 years and pregnant women with malaria in the Central African Republic, a ratio of reporters to households of 1:458 reflected that case management in people who sought out the CHW was their primary responsibility.^33^

**Table 2:** Ratios of surveillance reporters to population and households in order of increasing ratio

	**Event**	**Crisis phase**	**Setting**	**Reporters, n**	**Population, n**	**Ratio of reporters to population**	**Ratio of reporters to households**
Chad	Hepatitis E^35^	Fragile	Periurban	160	65 000	1:406	1:68
Chad	Mortality^19^	Acute	Camp	187*	93 227	1:499	1:83
Sierra Leone	Ebola virus diesase^32^	Fragile	Rural or remote	7416	3 981 665	1:537	1:89
Liberia	Multiple^44^	Fragile	Rural or remote	2972	2 065 690	1:695	1:100
Côte d'Ivoire	Multiple^24^	Fragile	Rural or remote	541	501 328	1:927	1:154
South Sudan	Polio^29^	Protracted	Rural or remote	3228	3 703 680	1:1147	1:191
Malawi	Mortality^45^	Acute	Camp	135*	269 859	1:1999	1:333
Central African Republic	Malaria^33^	Protracted	Rural or remote	80	220 000	1:2750	1:458
Côte d'Ivoire	Ebola virus disease^40^	Fragile	Rural or remote	110	421 448	1:3831	1:639
Guinea	Mortality^37^†	Fragile	Rural or remote	46	297 919	1:400	1:1079
Central African Republic	Mortality^21^†	Protracted	Rural or remote	24	158 000	1:450	1:1097
Democratic Republic of the Congo	Mortality^26^†	Protracted	Rural or remote	34*	311 295	1:9156	1:1526
Nepal	Mortality^38^	Acute	Camp	6	73 500	1:12 250	1:2042

* Inferred.

† Sentinel site surveillance.

For mortality systems, data were collected through household visits once a week or once a month (seven of ten systems) or passive reporting from communities (three of ten systems). During outbreaks, data collection was a mix of household visits once a week or once a month (four of ten systems) and passive reporting from the community (eg, so-called rumour surveillance) of suspect cases (two of ten systems) or events related to disease (two of 10 systems). CBS supplementing disease surveillance included household visits (three of six systems), often with passive notification of events related to disease or case detection based on consultations for malaria with the CHW. Both eradication programmes relied on so-called rumour surveillance from key informants.^27^, ^29^

Cases or events were often immediately reported via direct case referral to a designated health facility or by phone to the surveillance system, or both (13 of 25 systems). Reporting for outbreak detection was usually immediate (seven of ten systems). For mortality, reporting was done once a week (four of seven systems) or once or twice a month (three of seven systems).

Disease-focused CBS usually focused on a single disease that presented with salient symptoms and was familiar to the population (11 of 17 systems), including hepatitis E (presenting as acute jaundice syndrome), Guinea worm (presenting as skin lesion with emergence of a worm), or measles (presenting as fever and rash).^20^, ^23^, ^27^, ^28^, ^30^, ^35^ In protracted phases, fragile settings, and camps in Cox's Bazar, four systems focused on five to six epidemic-prone diseases (commonly, acute flaccid paralysis, measles, meningitis, and acute watery diarrhoea or cholera).^24^, ^41^, ^43^, ^44^ In acute-phase camp settings (eg, Bangladesh, Chad, the Democratic Republic of the Congo, Haiti, and Nepal), community mortality was a key event to monitor in anticipation of a deterioration of health and nutritional status or, uniquely in India, for birth and death registration of refugees.^19^, ^22^, ^31^, ^38^, ^41^ In India, Nepal, and Malawi, CHWs administered an additional questionnaire to another household member to ascertain the potential cause of death.^18^, ^38^, ^45^

In CBS of mortality in Guinea, the Democratic Republic of the Congo, and Malawi, mortality related to malaria and measles was detected by use of a community case definition (eg, mortality related to measles was defined as a death occurring ≤30 days after onset of rash, and not due to another clear cause, such as trauma) to enhance sensitivity of death and cluster detection in high-incidence contexts characterised by under-reporting.^30^, ^37^, ^45^ For the early detection of clusters, several CBS systems used events (eg, ≥2 ill people in a household; unexplained community deaths) for early identification of people with non-specific symptoms and as a lagging indicator of community transmission.^24^, ^32^, ^40^, ^44^

Systematic data management, verification, and analysis were rarely described. Data management and analytical approaches included the use of aggregation of paper registers once a week or once a month for tracking cases and transmitting data^26^, ^32^ and transfer of paper registers into software for automated analysis and reporting.^21^ Rarely, data were collected by use of mobile apps (eg, Côte d’Ivoire^24^, ^40^) or SMS (eg, Haiti and Sierra Leone).^36^, ^39^

Investigation and response to acute events was seldom described. Ebola virus disease events in Sierra Leone were first triaged and investigated by supervisors and then channelled into the district Ebola Response Centre alert system for investigation, alongside other alerts.^32^ In Cox's Bazar, Côte d’Ivoire, and Liberia, CHWs or CHVs notified local health-facility staff who were responsible for triaging, verifying, and investigating alerts and providing early responses.^24^, ^41^, ^44^ In eradication programmes in South Sudan, key informants triggered local supervisors and WHO field staff to promptly verify, investigate, and respond to suspect cases.^27^, ^29^

## Evaluation of surveillance system performance

### Multiple disease systems

In Liberia, an existing CBS system focused on Ebola virus disease was scaled to include 12 epidemic-prone diseases and community mortality for nearly 40% of the population in rural and remote areas.^44^ From April to September, 2016, although a low validity of case-based reporting (24% [885 of 3746] of alerts amounted to suspect cases) was noted, CBS was the source of 32% of all disease reporting. Positive predictive value was high for community mortality (ie, neonatal, 70%; maternal, 82%) and low for viral haemorrhagic fever, meningitis, and acute flaccid paralysis (8–14%). CBS at this scale amounted to 19% of government health expenditure and was judged to be financially unsustainable in its original format.

In Côte d’Ivoire, following the Ebola virus disease outbreak, a CBS system focused on Ebola virus disease^40^ was scaled to five epidemic-prone diseases and unusual health events in three districts bordering Guinea.^24^ CBS showed the ability to detect meaningful public health events in remote areas, although with low validity.^24^ From April, 2016, to December, 2017, 11% (420 of 3734) of signals amounted to suspect cases, although the proportion verified varied considerably (acute flaccid paralysis, 49% [33 of 68]; yellow fever, 29% [166 of 568]; measles, 18% [198 of 1097]; meningitis, 16% [23 of 144]; cholera, no signals). 23 of 420 (5%) suspect cases were laboratory-confirmed for measles, meningitis, or yellow fever. None of the nearly 5000 unusual events met the suspect event definitions to trigger investigation. The programme, although resource intensive, benefitted from mobile data collection and intensive supervision supported by non-governmental organisations.

In Cox's Bazar, Bangladesh, from May to November, 2019, a comprehensive evaluation of CBS, integrated with EWARS for five epidemic-prone diseases and community mortality, reported promising results for valid data and timely detection and response during a period of cholera and diphtheria outbreaks.^41^ CBS trends were consistent with health-facility surveillance. Positive predictive value was highest for acute flaccid paralysis, acute watery diarrhoea, and measles (74–100%), but lower for meningitis (50%) and diphtheria (42%). Alerts triggered an investigation team or response team, or both. Over the 6-month period, 21 positive rapid diagnostic tests for cholera triggered targeted responses. The integration of CBS with investigation and response teams was intensive relative to yield; CBS and the response teams required 354 staff yet similar alerts were also sourced from EWARS.

### Mortality systems

In refugee camps in Chad from 2004 to 2008, an evaluation of validity, simplicity, flexibility, and timeliness reported robust mortality surveillance with challenging issues for data validation.^19^ Although implemented in the acute phase, CBS took 2–5 months to scale and was judged to be too slow for immediate use, when reliable information was needed to establish the burden and causes of mortality. Interruption due to insecurity over the 4 years was minimal. Lower crude mortality rates and mortality rates for children younger than 5 years, detected through CBS compared with that of retrospective mortality surveys among similar populations, suggested that CBS under-reported deaths or overestimated population denominators, or both. In two documented instances, CBS detected high crude mortality rates or mortality rates for children younger than 5 years, which, when investigated, were related to acute increases in incidence of diarrhoeal disease, poor-quality water, and admissions for severe acute malnutrition.

For sentinel mortality surveillance in the Central African Republic from March to December, 2010, validity, sensitivity, and positive predictive value were high.^21^ A low proportion of household non-response was reported, considering the crisis context (ie, <1·0% (26 of 3969) refusal and 6·3% (252 of 3969) of households could not be contacted).^21^ There was minimal discordance between paper and electronic databases and no major interruptions to data collection. A capture–recapture analysis that used lists of deaths from three sources (ie, CBS, birth attendants, and religious leaders) showed that CBS data had sensitivity of 93% (compared with 52% from religious leaders and 29% from birth attendants) and positive predictive value of 91%.

During sentinel mortality surveillance from October, 2011, to September, 2012, in Fizi Health Zone in North Kivu, Democratic Republic of the Congo, high sensitivity, specificity, and positive predictive value were noted.^26^ An evaluation compared a retrospective mortality survey over the surveillance period and resolved differences between surveillance-tracked births and deaths. CBS showed improved sensitivity (87% *vs* 71%), specificity (>99% *vs* 99%), and positive predictive value (>91% *vs* 28%) compared with the survey. Data collection was reduced to a minimal dataset, where data collection could be deferred in case of insecurity. Validity was reinforced with multiple layers of data checking by supervisors. The fluid definitions of household, resident, and visitor, in addition to population movement for health care, affected accurate quantification of population denominators.

For mortality related to malaria in Guinea from July, 2011, to June, 2014, the response rate was 100%, showing high acceptability of CBS.^37^ During that period, deaths occurring at home increased from 69% to 80% and, in the health facility, decreased from 22% to 12%, showing the ability for CBS to fill the gap in the detection of community deaths. Deaths related to malaria represented 55·2% (685 of 1242) of all deaths (5·5% [68] of all deaths were retrospectively classified as related to Ebola virus disease). The first two deaths related to Ebola virus disease were the first laboratory-confirmed cases in the west African Ebola virus disease outbreak in 2014, showing the flexibility of CBS to rapidly detect emergent pathogens. Population movements were not documented to simplify data collection and, therefore, the effect of fluctuating population sizes could not be ascertained.

### Single disease systems

In Haiti, from 2014 to 2015, cholera CBS by Red Cross volunteers with mobile phones showed reliability through temporal trends that closely reflected that of routine surveillance.^39^ CBS trend data were investigated by the same stakeholders involved in routine surveillance, allowing for in-depth comparison of areas less covered by low sensitivity routine surveillance and better covered by CBS (eg, rural and disaster-affected areas).

In Sierra Leone, the national community event-based surveillance system for Ebola virus disease events, which also used mobile phones, was evaluated alongside routine Ebola virus disease surveillance from February to September, 2015.^32^, ^36^, ^42^ Of the 12 126 community event-based surveillance alerts produced, 86% (10 421) were for community deaths, 14% (1646) for illnesses, and less than 1% (7) for unsafe burials. 2% (287 of 12 126) of alerts met the suspect case definition and 16 of 287 suspect cases were confirmed. These findings produced a sensitivity of 30% (16 of 53 confirmed cases detected by routine Ebola virus disease surveillance during the study period), which complemented routine surveillance by identifying cases with no epidemiological link at detection. CBS produced continuous surveillance data in districts reporting few or no cases. CHWs detected a low proportion of illness and unsafe burials, suggesting low sensitivity of these events. CHWs reported good motivation among CHWs and CHVs to participate and that transmission of reports by SMS was feasible in rural settings.^36^, ^42^ Training and procurement of supplies was delayed until June, 2015, when the national outbreak was past its peak.

An evaluation from October, 2015, to September, 2017, of a large-scale CBS programme for poliomyelitis eradication in 34 counties of South Sudan affected by conflict reported higher sensitivity compared with health-facility surveillance (12·2 cases detected per month *vs* 2·2 cases per month).^29^ Compared with the preimplementation period, 92% (23 of 25) versus 64% (16 of 25) of counties reported consistently. Conversely, low stool sampling rates were reported for CBS (51–63% *vs* 100%) due to the scarcity of materials in remote areas and challenges with transport by the UN air service to the central laboratory.

## Findings from descriptive studies

Increased sensitivity of CBS for case finding compared with health facility-based surveillance was shown when CBS detected at least 90% of deaths related to measles in Katanga, the Democratic Republic of the Congo, during a large outbreak in 2011.^30^ CBS also maintained surveillance during crises where the use of health facilities was low or non-existent.^26^, ^29^, ^33^, ^46^ In the Central African Republic, malaria surveillance maintained close to 100% completeness of reporting throughout an acute crisis, decreasing to less than 60% after violence occurred in Bangui in 2013.^33^ Although insecurity continued to affect field movements, payments, supervision, and other core operations, CBS still provided the only malaria surveillance system throughout the crisis.

In refugee camps, CBS was used to identify the burden and investigate potential causes of mortality to drive preventive measures. On the basis of mortality findings through CBS in camps, in Nepal in 1992, CHWs did case finding, management, and referral and early detection and response for cholera, *Shigella dysenterae*, and meningitis outbreaks.^38^ Similarly, CBS in camps in Malawi in 1987–89 aided in monitoring temporal changes in suspected mortality from preventable and treatable diarrhoea, malaria, acute respiratory illness, and measles in children younger than 5 years.^45^ Among Tibetan refugees in India, from 1994 to 1996, CBS showed that infant mortality was lower than that of the host population, and enabled investigation of trends in cause-specific mortality.^18^

Some studies suggested waning acceptability by community members. In the Democratic Republic of the Congo in 2011–12, families reported fatigue of being repeatedly asked about mortality, and fatigue among CHWs was suspected as well.^26^ Similarly, CBS was scaled down in postearthquake Haiti in 2010 due to community frustration with visits once per week, which did not offer direct benefit, leading community members to threaten surveillance workers.^31^

## Discussion

This Review suggests that, during crises, CBS can provide sensitive disease-detection capacity, produce valid disease and mortality data for early warning of events and deteriorations in health status, rapidly target interventions to populations who are at risk, and operate during cycles of insecurity. However, there are logistical, financial, and technical costs. Continuous supervision for large community networks, and sufficient human resources to verify a typically high proportion of signals to valid events, are required. The community workforce has to absorb surveillance into existing responsibilities, which might lead to waning motivation and decreasing data quality, past the acute crisis period.

Key elements for effective CBS systems appear to include a small set of diseases under surveillance,^21^, ^29^, ^39^, ^41^ use of syndromic case definitions,^30^, ^41^ integration of the system among CHWs and CHVs within communities,^24^, ^26^, ^32^, ^36^, ^43^ and efficient use of the existing investigation and response capacity.^24^, ^29^, ^32^, ^36^, ^44^ Improved disease detection appears to be related to several factors. First, the low positive predictive value of most of the 12 diseases and events in Liberia might have been improved with a smaller list of the most immediate threats that would be better recognised by CHWs.^44^ Syndromic case definitions for diseases that have relatively specific symptoms and are familiar to communities should be more successful (eg, measles and hepatitis E) than diseases with less specific symptoms (eg, meningitis) or a large number of events. Deaths can provide a specific, albeit lagging, indicator of an outbreak or deterioration of population health. Second, as recognised by the International Federation of Red Cross and Red Crescent Societies, CBS functions in the presence of a social contract between the community who agree to report events in exchange for an appropriate response from the implementing agency, or other clear benefits.^12^ Participants should be regularly assured of the benefits through community engagement and timely, effective responses.^10^ The importance of a clearly understood social contract can be seen in the waning acceptability of CBS in Haitian camps due to imperceptible benefit, or the apparent willingness to report deaths in Nepalese camps when a burial shroud was given to facilitate a dignified burial.^31^, ^38^ Third, CBS requires efficiency as a driving principle, given the high volume of signals for verification.^47^ Verification should be internal (eg, via CBS supervisors). Verified events should then be integrated into the investigation and response functions of EWARS or the health system to ensure consistency and efficient use of scarce human and material resources.^10^, ^24^, ^41^

Strong training, supervision, and feedback mechanisms need to be maintained to support large community networks and integrated within the existing disease surveillance and response infrastructure, if available.^10^, ^19^, ^21^, ^27^, ^32^, ^33^, ^39^ Financial and material incentives most likely influence a CHW's ownership of the role, and in turn, timely collection of data and programme longevity. Incentives were insufficiently discussed in these articles but have been identified across agencies as a key implementation challenge.^10^ CBS consistently brings a voluminous workload for CHWs, CHVs, and Red Cross networks. Exit strategies were rarely discussed, but several authors called into question the sustainability of CBS beyond acute crises and outbreaks due to the need for external support from non-governmental organisations and the mounting responsibilities of CHWs and CHVs.^24^, ^26^, ^32^, ^41^, ^43^, ^44^ Other options, such as the integration of CBS functions for existing CHW, CHV, and Red Cross networks for temporary use during acute crises and outbreaks, or passive detection of events that requires few resources,^41^ could represent more efficient approaches. Similarly, where disease trends are of primary interest, implementation of sentinel site surveillance in a small area should be considered, rather than exhaustive detection, where an elimination strategy is pursued (eg, for Ebola virus disease and poliomyelitis).^21^, ^26^, ^37^, ^48^

Once running, CBS systems benefit from early and routine evaluation. At the minimum, an evaluation should occur within the first few months of operation and include an evaluation of community willingness to participate and an assessment of data validity (eg, CHW or CHV recall of case definitions and accuracy of population estimation), positive predictive value and sensitivity (eg, proportion of case definitions correctly applied by CHWs or CHVs; contribution of CBS relative to overall surveillance), and population coverage.^32^, ^36^ The timeliness of the system for the pipeline for notification, investigation, and analysis should be assessed to address how data can be used in real time for public health action and advocacy.^41^

CBS implementation should also benefit from advances in electronic tools for surveillance and for managing the data pipeline.^49^, ^50^, ^51^ Despite most of the systems being established in the past decade, only four systems reported the use of mobile apps or SMS for data collection.^24^, ^36^, ^39^, ^40^ Mobile phones and smartphones have increased timeliness and completeness of surveillance reporting by health workers in the Central African Republic and Papua New Guinea, which are affected by conflict and fragility, and through mobile EWARS in Cox's Bazar and northern Nigeria.^52^, ^53^, ^54^, ^55^ However, for CBS, it is imperative that mobile apps be purpose built for use by community members who might be unfamiliar with mobile phones.^56^ Particular to crisis settings, several challenges exist, including poor cellular penetration in remote areas^57^ and the costs of providing infrastructure (eg, telephone fleet, solar chargers, and software development) where it does not exist or has been disrupted.^24^, ^36^, ^49^, ^50^, ^52^ A key ethical consideration is the array of benefits (eg, obviating household visits via telephone calls or SMS) and risks (eg, confidential data collected on a mobile phone, particularly for mortality) that electronic data collection brings during conflict.^56^, ^58^, ^59^ Building on improved electronic data collection and management,^51^ statistical techniques used in syndromic surveillance to model detection algorithms that separate signal (ie, true positives) from noise (ie, false positives) should also be explored to help to triage by alert type and reduce unmanageable workloads.^60^

Our Review is subject to limitations. The 25 systems discussed here might not be fully generalisable across contexts. We relied primarily on published articles, whereas organisations that carry out CBS, such as International Federation of Red Cross and Red Crescent Societies, do not habitually contribute to these forums. Most reports described CBS in camps and rural settings, but there were few reports of CBS in urban settings, or in level-3 humanitarian crises, where poor access and disrupted health systems frequently result in poor detection of outbreaks.^61^, ^62^, ^63^ Varying standardisation of CBS designs and performance indicators did not allow for comparison of different systems.

**Search strategy and selection criteria**

Included articles described or evaluated community-based surveillance for communicable disease or mortality, or both, delivered by community health workers or volunteers, Red Cross society volunteers, or community key informants, in countries that were fragile or affected by conflict. We searched PubMed, MEDLINE, and Embase databases in a general search (search 1) and a country-specific search (search 2) between July 20, 2020, and Aug 10, 2020. Search 1 terms included “community surveillance”, “community event-based surveillance”, “community-based surveillance”, “community-based mortality surveillance”, (“community health worker” AND surveillance), and (“community volunteer” AND surveillance). Search 2 terms combined any of: “population surveillance”, “infectious disease surveillance”, “communicable disease surveillance”, “public health surveillance”, “sentinel surveillance”, “communicable disease control*”, “disease outbreaks”, “epidemics”, “community volunteer”, or “community health worker”, WITH the name of eligible fragile and conflict-affected countries. Epidemiologists at major humanitarian organisations that implement CBS in crises (eg, International Federation of Red Cross and Red Crescent Societies, CARE, International Rescue Committee, and Médecins Sans Frontières) were emailed to seek grey literature. References of retrieved articles and an additonal scoping review of CBS in low-income and middle-income countries were searched. Abstracts were included given the novelty of the subject. All study designs were eligible and there were no date or language restrictions. Excluded articles included those on surveillance not pertaining to communicable diseases (eg, nutrition, non-communicable diseases, or animal surveillance).

## Conclusion

Early detection and response to outbreaks and deteriorations in the health status of a population are priorities for surveillance in populations affected by crises. Our Review provides a case for CBS as a crucial component of surveillance throughout the acute to protracted crisis phases and in countries with disrupted public health systems. Like other community health systems, however, extensive planning of objectives and for adequate resources, technical assistance, supervision, monitoring, and evaluation will mediate the effectiveness of CBS.
