# Supplementary material for: People-centred surveillance: a narrative review of community-based surveillance among crisis-affected populations
Source: Lancet Planet Health. 2020 Oct 7;4(10):e483–95. doi: 10.1016/S2542-5196(20)30221-7 (PMC7542093; doi:10.1016/S2542-5196(20)30221-7)
Supplement: Supplementary appendix [file mmc1.pdf]

THE LANCET
Planetary Health

Supplementary appendix

This appendix formed part of the original submission and has been peer reviewed. We post it as supplied by the authors.

Supplement to: Ratnayake R, Tammaro M, Tiffany A, Kongelf A, Polonsky JA, McClelland A. People-centred surveillance: a narrative review of community-based surveillance among crisis-affected populations. *Lancet Planet Health* 2020; **4:** e483–95.

People-centred surveillance: a narrative review of community-based surveillance among crisis-affected populations

Fragile and conflict-affected countries……………………………………………..………..p 1

Search terms and results……………………………………………………………..…...p 2

Fragile and conflict-affected countries

The World Bank's classification of fragile and conflict-affected countries uses the following definitions[1]:

1. Countries with high levels of institutional and social fragility, identified based on publicly available indicators that measure the quality of policy and institutions and manifestations of fragility.

2. Countries affected by violent conflict, identified based on a threshold number of conflict-related deaths relative to the population. This category includes two sub-categories based on the intensity of violence: countries in high-intensity conflict and countries in medium-intensity conflict.

Countries that appeared on the annual list more than half of the time during the 2008-2019 period (e.g. 6/12 years) were assessed to be sufficiently fragile and/or conflict-affected, and were included in Search 2 of the review. Small states were excluded (Comoros, Kiribati, Marshall Islands, Micronesia, Fed. Sts., Solomon Islands, Tuvalu).

The final list of fragile and conflict-affected states included:

**WHO region[2]**	**Countries**
Africa	Angola, Burundi, Central African Republic, Chad, Côte d'Ivoire, Democratic Republic of the Congo, Republic of Congo, Eritrea, The Gambia, Guinea, Guinea Bissau, Liberia, Mali, Sierra Leone, South Sudan, Togo, Zimbabwe
Americas	Haiti
Eastern Mediterranean	Afghanistan, Djibouti, Iraq, Libya, Somalia, Sudan, Syria, West Bank and Gaza/Palestinian Territories, Yemen
Europe	Bosnia and Herzegovina, Kosovo
South-East Asia	Myanmar, Timor-Leste
Western Pacific	--

Search terms and results

Search 1: General search

PubMed	((((((community surveillance[Title/Abstract] OR community event-based surveillance[Title/Abstract]) OR (community-based surveillance[Title/Abstract]) OR (community-based surveillance[Title/Abstract]) OR "community-based mortality surveillance"[Title/Abstract]) OR ((("community health worker[Title/Abstract]") AND surveillance[Title/Abstract])) OR (("community volunteer"[Title/Abstract]) AND surveillance[Title/Abstract])))))	451 results
EMBASE	1 "community based surveillance".ti,ab. (259)<br>2 "community surveillance".ti,ab. (284)<br>3 "community event based surveillance".ti,ab. (6)<br>4 "community based health surveillance".ti,ab. (8)<br>5 "community based mortality surveillance".ti,ab. (5)<br>6 1 or 2 or 3 or 4 or 5 (541)	541 results

Search 2: Country-specific search

PubMed	((((((((("population surveillance" [mh]) OR ("infectious disease surveillance"[Title/Abstract]) OR "communicable disease surveillance"[Title/Abstract] OR ("public health surveillance" [mh]) OR ("sentinel surveillance" [mh]) OR (communicable disease control*[mh]) OR ("disease outbreaks" [mh]) OR (epidemics [mh]) OR ("community volunteer"[Title/Abstract]) OR ("community health worker" [mh])))<br>AND<br>(Angola[Title] OR Afghanistan[Title] OR "Bosnia and Herzegovina"[Title] OR Burundi[Title] OR "Central African Republic"[Title] OR Chad[Title] OR "Cote d'Ivoire"[Title] OR "Ivory Coast"[Title] OR "Democratic Republic of Congo"[Title] OR "Republic of Congo"[Title] OR Congo[Title] OR Djibouti[Title] OR Eritrea[Title] OR Gambia[Title] OR Guinea[Title] OR "Guinea Bissau"[Title] OR Haiti[Title] OR Iraq[Title] OR Kosovo[Title] OR Liberia[Title] OR Libya[Title] OR Mali [Title] OR Myanmar[Title] OR "Sierra Leone"[Title] OR Somalia[Title] OR "South Sudan"[Title] OR	737 results

	Sudan[Title] OR Syria[Title] OR “Timor-Leste”[Title] OR Togo[Title] OR “West Bank and Gaza”[Title] OR “Palestinian Territories”[Title] OR Yemen[Title] OR Zimbabwe[Title] OR refugee*[Title])<br>AND<br>(("warfare and armed conflicts" [mh]) OR ("armed conflicts" [mh]) OR ("warfare" [mh]) OR ("disasters" [mh]) OR (refugees[mh]) OR ("emergencies" [mh]) OR (relief work*[mh])))))))	
EMBASE	1 "population surveillance".ti,ab. (335)	258 results
	2 "public health surveillance".ti,ab. (2157)	
	3 "sentinel surveillance".ti,ab. (1626)	
	4 "communicable disease control".ti,ab. (425)	
	5 "communicable diseases".ti,ab. (10951)	
	6 "disease outbreaks".ti,ab. (4623)	
	7 "epidemics".ti,ab. (26373)	
	8 "community networks".ti,ab. (406)	
	9 "community health workers".ti,ab. (4482)	
	10 "warfare and armed conflicts".ti,ab. (1)	
	11 "armed conflicts".ti,ab. (461)	
	12 "warfare".ti,ab. (5692)	
	13 "disasters".ti,ab. (11397)	
	14 "refugees".ti,ab. (8156)	
	15 "emergencies".ti,ab. (27884)	
	16 "relief work".ti,ab. (147)	
	17 Angola.ti,ab. (1629)	
	18 Afghanistan.ti,ab. (7042)	
	19 Burundi.ti,ab. (908)	
	20 Central African Republic.ti,ab. (1131)	
	21 Chad.ti,ab. (1452)	
	22 Cote d'Ivoire.ti,ab. (2559)	
	23 Ivory Coast.ti,ab. (2050)	
	24 Democratic Republic of Congo.ti,ab. (2227)	
	25 Djibouti.ti,ab. (442)	
	26 Eritrea.ti,ab. (675)	
	27 Gambia.ti,ab. (2591)	
	28 Guinea.ti,ab. (144398)	
	29 Guinea Bissau.ti,ab. (1106)	
	30 Haiti.ti,ab. (3872)	
	31 Iraq.ti,ab. (10118)	
	32 Kosovo.ti,ab. (1260)	
	33 Liberia.ti,ab. (1824)	
	34 Libya.ti,ab. (1548)	
	35 Mali.tb,ab. (3797)	

36	Myanmar.ti,ab. (3803)
37	Republic of Congo.ti,ab. (2644)
38	Sierra Leone.ti,ab. (2570)
39	Somalia.ti,ab. (1491)
40	South Sudan.ti,ab. (622)
41	Sudan.ti,ab. (10629)
42	Syria.ti,ab. (2421)
43	Timor-Leste.ti,ab. (397)
44	Togo.ti,ab. (1703)
45	Palestinian Territories.ti,ab. (135)
46	Yemen.ti,ab. (2154)
47	Zimbabwe.ti,ab. (5947)
48	refuge*.ti,ab. (15193)
49	West Bank.ti,ab (803)
50	Gaza.ti,ab (1266)
51	49 and 50 (257)
52	Bosnia.ti,ab. (2957)
53	Herzegovina.ti,ab. (2295)
54	52 and 53 (2238)
55	17 or 18 or 19 or 20 or 21 or 22 or 23 or 24 or 25 or 26 or 27 or 28 or 29 or 30 or 31 or 32 or 33 or 34 or 35 or 36 or 37 or 38 or 39 or 40 or 41 or 42 or 43 or 44 or 45 or 46 or 47 or 48 or 51 or 54 (227491)
56	1 or 2 or 3 or 4 or 5 or 6 or 7 or 8 or 9 (50362)
57	10 or 11 or 12 or 13 or 14 or 15 or 16 (52572)
58	55 and 56 and 57 (258)

References

1. World Bank. Classification of Fragile and Conflict-Affected Situations. July 9, 2020 2020. https://www.worldbank.org/en/topic/fragilityconflictviolence/brief/harmonized-list-of-fragile-situationsJuly 20, 2020).
2. World Health Organization. Alphabetical List of WHO Member States. 2020. https://www.who.int/choice/demography/by_country/en/ (accessed July 20, 2020).
